# Root hair initiation-dependent and -independent nuclear movements during root hair development in *Arabidopsis thaliana*

**DOI:** 10.1080/15592324.2026.2726829

**Published:** 2026-09-17

**Authors:** Hirotomo Takatsuka, Chisako Yasuda

**Affiliations:** a Department of Biological Sciences, Graduate School of Humanities and Sciences, Nara Women's University, Kitauoya-Nishimachi, Nara, Nara, Japan

**Keywords:** Root hair, nuclear migration, nuclear movement, actin, ROOT HAIR DEFECTIVE 6 (RHD6), root hair initiation

## Abstract

Nuclear migration toward the tip is an essential intracellular structural reorganization that drives tip growth in root hairs. Previous studies have demonstrated that the nucleus is delivered to the root hair tip via three sequential steps. However, it remains poorly understood what triggers each step. Here, we show that the 1st step—the shift from the cell center toward the inner lateral membrane of root hair precursor cells—normally occurs in root hair precursor cells lacking ROOT HAIR DEFECTIVE 6 (RHD6)-induced root hair initiation, which is characterized by hair bulge formation. In contrast, the 2nd step—migration from the inner lateral membrane toward the root hair initiation site—is abolished in RHD6-deficient cells, indicating that the 1st and 2nd steps represent root hair initiation-independent and -dependent nuclear movements, respectively. Unlike the subsequent steps, the 1st step occurs normally even in the absence of the major actin genes, namely ACTIN2 and ACTIN7, raising the possibility that an actin-independent force steers the 1st step of nuclear migration in root hair cells. Furthermore, undergoing the inward movement of the first step is unexpectedly not mandatory to proceed to the next stage. Altogether, our findings highlight the existence of elaborate yet flexible mechanisms that ensure precise nuclear delivery to its destination in root hairs.

## Introduction

Root hairs are single-celled tubular structures that extend from the root epidermis. By significantly increasing the root surface area, they enhance the uptake of water and nutrients from the soil, thereby promoting overall plant growth.^1^ ^,^ ^2^ The functional importance of these structures is underscored by the fact that plants with shortened root hairs exhibit reduced biomass under nutrient-limited conditions.^3^

Root hairs elongate via tip growth, a process where cell wall synthesis and expansion are confined at the very apex.^4^ ^,^ ^5^ To sustain this active and localized growth, root hairs undergo dramatic structural rearrangements at the intracellular level. One of the most striking examples is nuclear movement toward the tip of growing root hairs. Because trapping the nucleus with optical tweezers prematurely arrests root hair elongation, nuclear migration toward the tip is considered a prerequisite for continuous root hair growth.^6^

**Figure 1.**
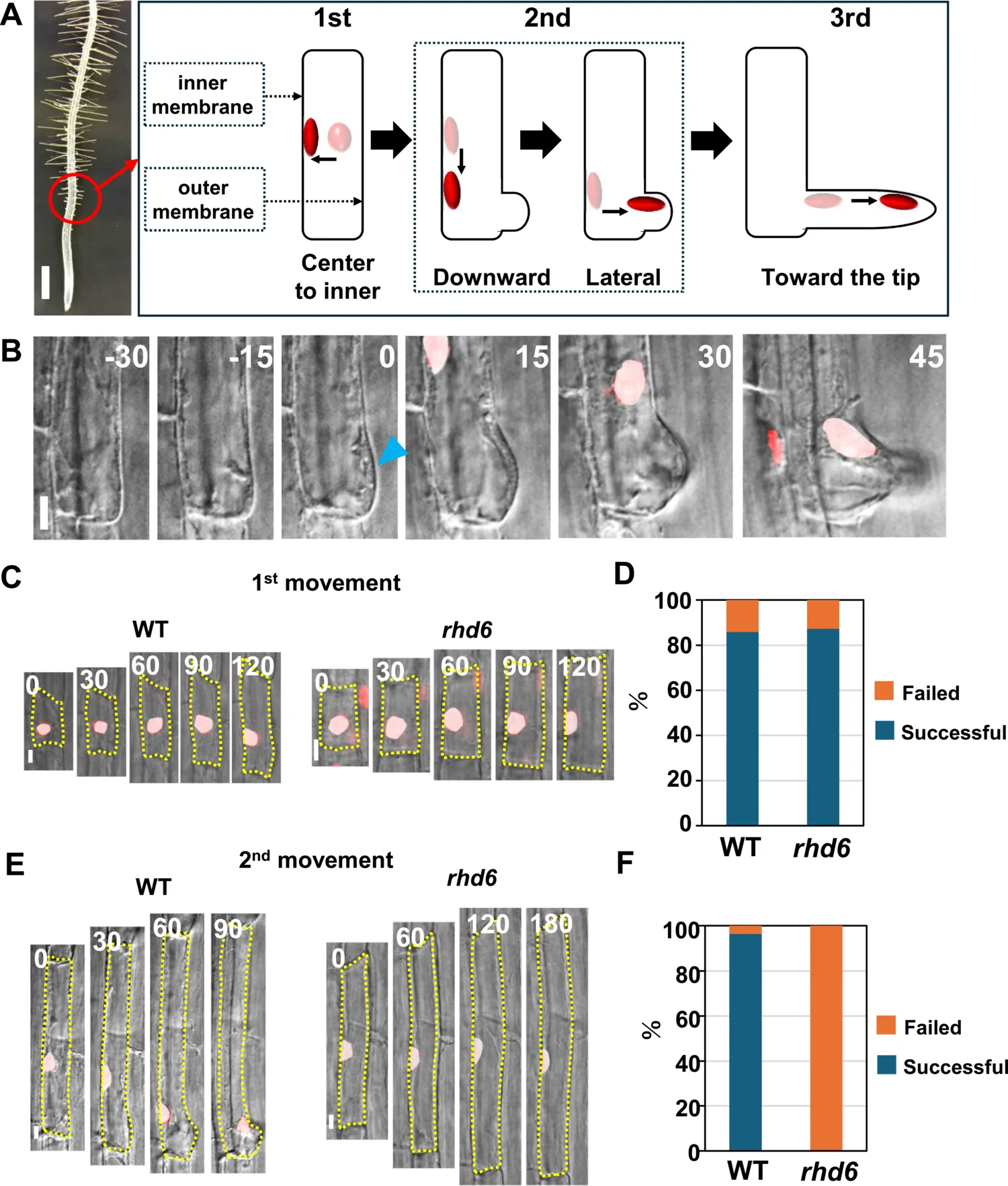
Root hair initiation is required for the 2nd, but not the 1st step of nuclear migration. (a) A schematic illustration of nuclear migration during root hair development. The scale bar represents 1 mm. (b) Representative images of a root hair precursor cell undergoing nuclear movement in five-day-old roots of wild-type carrying *H2B-tdTomato*. Numbers shown in the panels indicate the time (min). Time 0 indicates the moment when the root hair bulge first became visible. The blue arrowhead represents the bulged root hair. Nuclei are shown in white. The scale bar represents 10 µm. (c and d) Root hair precursor cells undergoing the 1st (c) or 2nd (d) step of nuclear migration in five-day-old wild-type (WT) and *rhd6* mutant roots carrying *H2B-tdTomato*. Yellow broken lines and numbers indicate the cell outlines and time (min), respectively. Time 0 refers to the beginning of the 1st step. Nuclei are shown in white. The scale bar represents 10 µm. (e and f) The percentage of root hair cells that failed or completed the 1st (e) or 2nd (f) step. *N* ≧ 48.

Based on previous studies, nuclear migration prior to the cessation of root hair growth can be divided into three distinct steps^7^ ^,^ ^8^: (1) The nucleus shifts from the cell center toward the inner lateral membrane of root hair precursor cells (1st step); (2) The nucleus migrates toward the root hair initiation site (RHIS), which is established near the basal end of the outer membrane of root hair precursor cells (2nd step); and (3) Following the onset of root hair outgrowth, the nucleus tracks behind the elongating tip, maintaining a constant distance from the apex (3rd step) (Figure 1A). Recently, we discovered that the 2nd step toward the RHIS is further divided into two sequential movements: an initial downward movement, followed by a lateral movement^8^ (Figure 1A). Our findings demonstrated that the downward movement is driven by filamentous actin (F-actin) composed of two distinct types of actin isoforms, ACTIN7 (ACT7) and ACTIN2 (ACT2)/ACTIN8 (ACT8). In contrast, the subsequent lateral movement is facilitated by F-actin predominantly utilizing ACT2/ACT8 as the globular actin (G-actin) source, assembled in a ROP-dependent manner.^8^

Despite these findings, two critical questions regarding the 2nd step of nuclear migration remain open: (1) is it mandatory for the nucleus to relocate toward the inner lateral membrane prior to the onset of the 2nd step? and (2) what cues trigger the 2nd step following the completion of the 1st step?

Here, we show that the 1st movement is not affected in the mutant of a basic helix–loop–helix transcription factor ROOT HAIR DEFECTIVE 6 (RHD6) that acts as a master regulator of root hair initiation. In contrast, the 2nd step is completely abolished in RHD6-deficient cells, indicating that the 1st and 2nd steps are root hair initiation-independent and -dependent nuclear movements, respectively. Unlike the 2nd and 3rd steps, the 1st step occurs normally even in the absence of ACT2 and ACT7, raising the possibility that the 1st step is steered in an actin-independent manner. Furthermore, the 1st step is unexpectedly not mandatory to proceed to the next step. Altogether, our findings reveal the unique mechanisms to precisely deliver the nucleus to its destination in root hairs.

## Materials and methods

### Plant materials and growth conditions

*Arabidopsis thaliana* (ecotype Col-0) was grown vertically on Murashige and Skoog (MS) plates [1× MS salts, 0.5 g/l 2-(*N*-morpholino)ethanesulfonic acid (MES), 1× MS vitamin solution, 1% sucrose, and 0.4% phytagel (pH 6.3)] under continuous light conditions at 23°C^9^. The *act7* (*act7-6*), *act2/8* (*act2-1 act8-2*), *act2/7* and the transgenic line *H2B-tdTomato* (*pRPS5A:H2B-tdTomato*) were previously described.^10–12^ The *rhd6* (*rhd6-1*) knockout mutant was kindly provided by Dr. Michitaro Shibata (RIKEN, Japan).

### Microscopic observation

Seedlings were transferred to the glass-bottom dish (Iwaki) and were gently covered with solid MS medium. After a 1 h incubation, the samples were placed on the stage of an inverted fluorescence microscope (Eclipse Ti2, Nikon) equipped with a confocal laser scanning unit (A1 HD25, Nikon) and a 40× water immersion objective lens. Confocal images of the median plane of the root hair cells were captured at 15-min intervals until the nucleus completed the 1st or 2nd steps of nuclear migration.

Primary roots were photographed using a stereomicroscope (SZX7, EVIDENT) equipped with a CMOS camera (AdvanCam-HD2sA, AdvanVision) at a total magnification of 8×.

### DAPI staining

Five-day-old wild-type and 14-d-old *act2/7* double mutant seedlings were fixed in a methanol:acetic acid (3:1, v/v) solution for 4 h at room temperature, followed by washing with water. The samples were then incubated in PBS buffer containing 2 µg/mL 4′,6-diamidino-2-phenylindole (DAPI) for 30 min and subsequently washed with PBS buffer three times. Finally, the roots were mounted in PBS buffer and observed using a confocal laser scanning microscope.

## Results and discussion

The formation of a root hair bulge, a swelling at the distal end of the epidermal cell, marks the very first step in root hair development and can be easily identified using bright-field microscopy.^13^ ^,^ ^14^ While we recently unveiled the unique nature of the 2nd step of nuclear migration during root hair development,^8^ the temporal relationship between the 2nd step of nuclear migration and bulge formation remains to be examined. Here, we conducted quantitative analyses to determine whether the 2nd step occurs in root hair cells that have already bulged, or if the nucleus can migrate to the future RHIS without obvious bulge formation. The results showed that in all wild-type hair cells tested, the nucleus arrived at the RHIS following bulge formation (*n* = 100) (Figure 1B). By contrast, in those rare instances where wild-type hair cells failed to form a bulge (8.3%; 9 out of 109 cells), none of the nuclei underwent the 2nd step of nuclear migration, failing to reach the RHIS (Supplementary Figure 1). This implies that bulge formation is an indispensable event for the onset of the 2nd step. It should be noted that even in these bulge-less cells, the nucleus was located adjacent to the inner membrane, suggesting that the 1st step occurs independently of bulge formation.

To further test the requirement of bulge formation for the 1st and 2nd steps of nuclear migration, we examined the knockout mutants of *ROOT-HAIR DEFECTIVE 6* (*RHD6*) (Figure 1C–F). RHD6 is a basic helix–loop–helix transcription factor that acts as a master regulator of root hair initiation in *A. thaliana.* ^15^ In *rhd6* hair precursor cells, which properly acquire hair cell fate but fail to form a root hair bulge, the nucleus completed the 1st step, but did not initiate the 2nd step, remaining at the cell center adjacent to the inner membrane (Figure 1C–F). These results reinforce the idea that root hair initiation (characterized by bulge formation), where RHD6 acts as a key regulator, is not a prerequisite for the 1st step but is indispensable for the onset of the 2nd step of nuclear migration. Alternatively, RHD6 might have a function in triggering the onset of the 2nd step, independently of bulge formation. Previously conducted transcriptome analyses have suggested that RHD6 regulates a diverse set of genes that control cellular processes required for root hair initiation, such as cell wall modification, vesicular trafficking, and cytoskeletal rearrangement.^16^ To date, however, direct target genes of RHD6 remain largely unidentified. Comprehensive identification of these target genes and their involvement in the second nuclear migration will help dissect the genetic pathways that trigger the onset of the 2nd nuclear migration.

**Figure 2.**
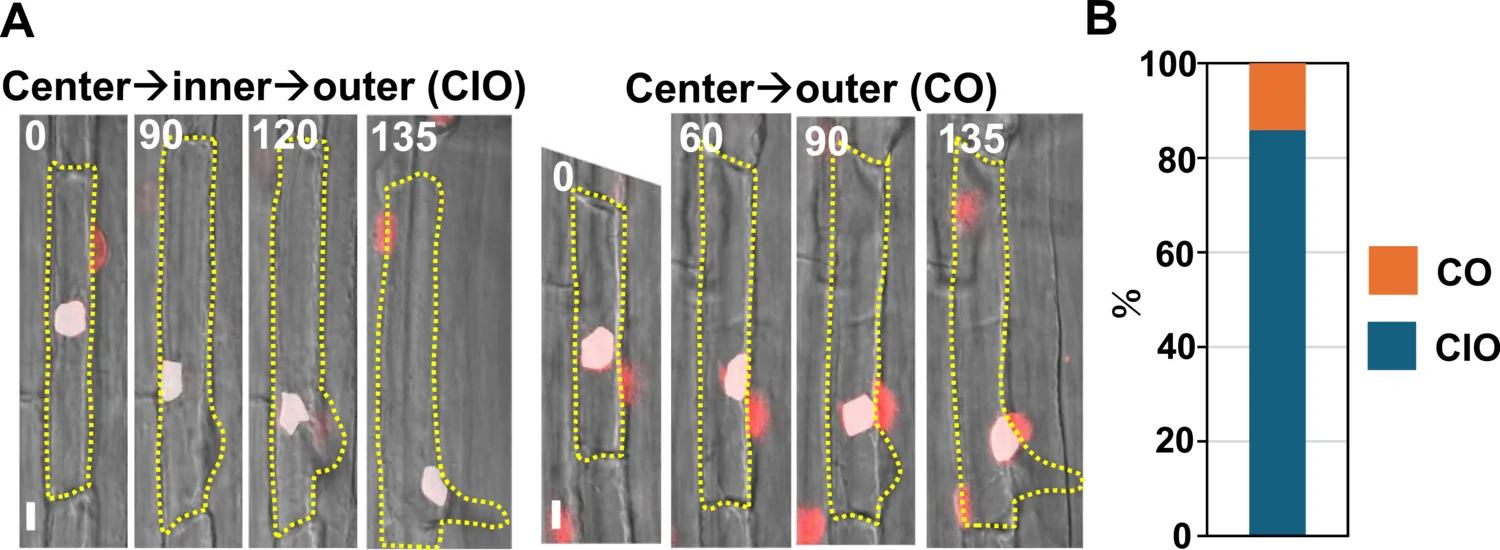
The inward nuclear movement is not mandatory for the nucleus to reach the root hair initiation site. (a) Representative images of root hair precursor cells undergoing CIO-mode (with inward movement) or CO-mode (without inward movement) nuclear migration in five-day-old wild-type roots carrying *H2B-tdTomato*. Yellow broken lines and numbers indicate the cell outlines and time (min), respectively. Time 0 refers to the beginning of the 1st step. Nuclei are shown in white. The scale bar represents 10 µm. (b) The percentage of cells showing inward (CIO) versus no inward (CO) nuclear movement among those that successfully migrated to the root hair initiation site. *N* = 106.

The 1st step toward the inner membrane causes the nucleus to detour toward the RHIS, which forms at the outer membrane. Our next question is whether this inward movement is mandatory for the nucleus to successfully migrate to the RHIS. To address this, we performed time-lapse imaging of wild-type root hair precursor cells and analyzed the trajectories of successfully migrated nuclei. The results showed that approximately 86% of the root hair nuclei underwent the inward movement (the CIO mode in Figure 2), whereas 14% of the nuclei that successfully migrated to the RHIS did not exhibit the inward movement prior to reaching their destination (the CO mode in Figure 2). These results suggest that the 1st step toward the inner membrane is not essential for nuclear migration toward the root hair tip. Nonetheless, given that the majority of root hair nuclei undergo this detour (Figure 2B), it is reasonable to assume that the 1st step toward the inner membrane carries an as-yet-unidentified physiological significance. Because the nucleus moves downward along the inner membrane after the 1st step (Figure 1A), one possible explanation is that approaching the destination (i.e., the RHIS) from the side opposite to the root surface may serve as a strategy to protect the nucleus from damage caused by soil pressure. It will be of great interest to examine how artificially applied pressure from the root surface influences nuclear dynamics in root hair cells exhibiting the CO mode nuclear migration.

**Figure 3.**
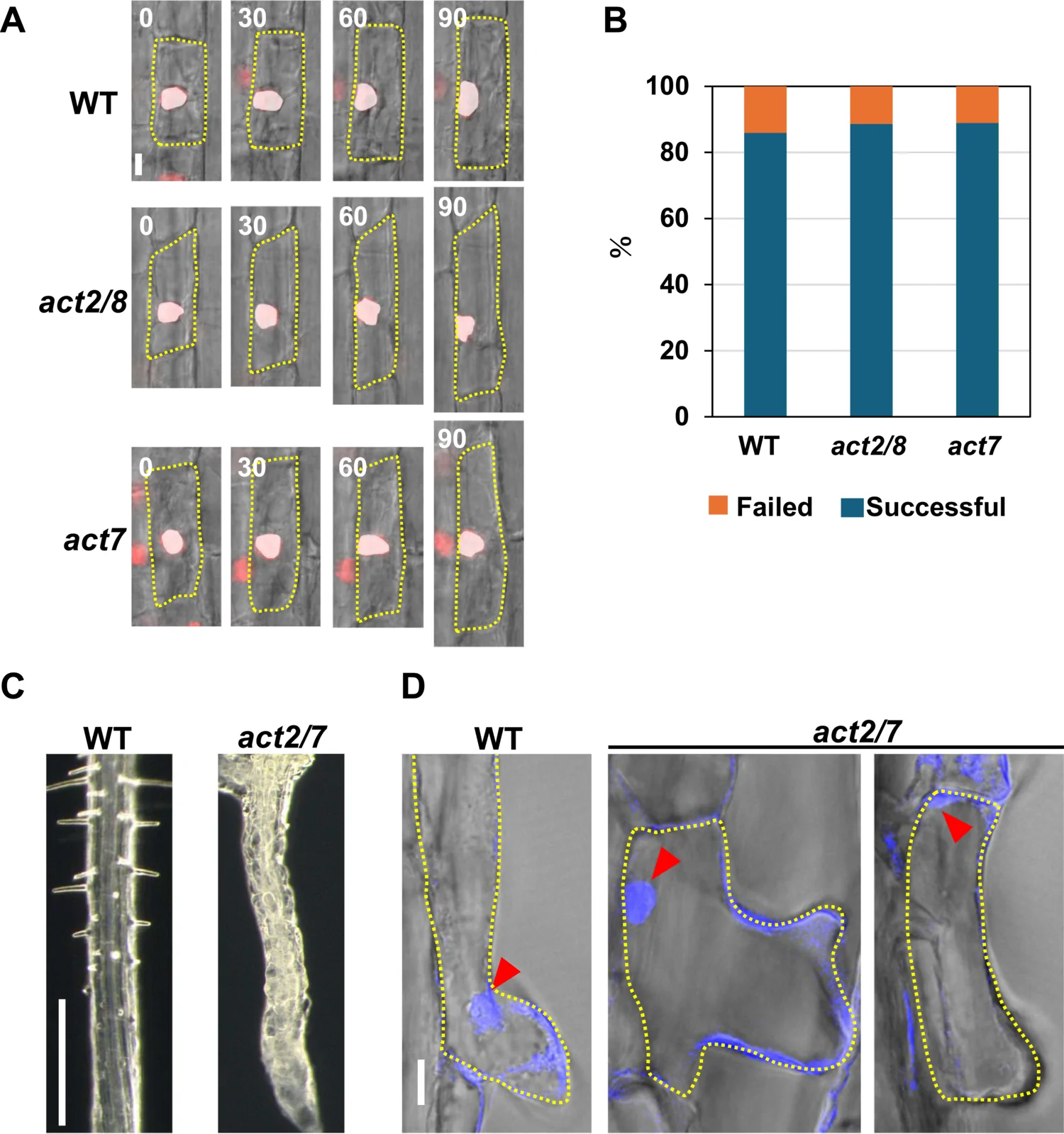
The 1st step of nuclear migration normally occurs in *act* mutants. (a) Root hair precursor cells undergoing the 1st step of nuclear migration in five-day-old wild-type (WT), *act2/8,* and *act7* mutant roots carrying *H2B-tdTomato*. Yellow broken lines and numbers indicate the cell outlines and time (min), respectively. Time 0 refers to the beginning of the 1st step. Nuclei are shown in white. The scale bar represents 10 µm. (b) The percentage of root hair precursor cells that failed or completed the 1st step. *N* ≧ 54. (c) Representative images of five-day-old roots of WT and 14-d-old *act2/7* double mutants. The scale bar represents 500 µm. (d) Bulged root hair cells in DAPI-stained five-day-old roots of WT and *act2/7* double mutants. Yellow broken lines and red arrowheads indicate the cell outlines and the position of nuclei, respectively. The scale bar represents 10 µm.

In plants, actin, rather than microtubules (MTs), serves as the primary driving force behind nuclear movement.^17^ ^,^ ^18^ While previous studies have clearly demonstrated that both the 2nd and 3rd steps of nuclear migration in root hair cells are actin-dependent,^6^ ^,^ ^8^ it remains unclear whether the 1st step also requires actin. Therefore, we investigated whether actin deficiency, caused by mutations in G-actin genes, impairs the 1st step. The *Arabidopsis* genome encodes three genes for vegetative G-actin, which are further divided into two sub-groups: *ACTIN 7* (*ACT7*) and *ACT2/ACT8.* ^10^ A previous study showed that the nuclear positioning prior to the 2nd step was almost identical between the wild-type and the *act7-6* loss-of-function mutant (hereafter referred to as *act7*),^7^ ^,^ ^8^ implying that G-actin encoded by *ACT7* is not crucial for the 1st step. Supporting this view, our live-imaging-based analyses revealed that *act7* nuclei moved normally toward the inner membrane, with a success rate comparable to that of the wild-type (Figure 3A and B). Likewise, the nuclei of the *act2-1 act8-2* double mutant (hereafter referred to as *act2/8*) underwent the 1st step as frequently as those of wild-type (Figure 3A and B), indicating that *ACT2* and *ACT8* are not crucial for the 1st step, either.

One possible interpretation for these results is that a small amount of G-actin, supplied from intact *ACT* genes, was sufficient to trigger the 1st step in both *act7* and *act2/8* mutants. To test this possibility, we attempted to generate *act2/7/8* triple mutants. Unfortunately, we were unable to obtain them, likely due to their essential roles in vegetative growth, leading us to examine *act2 act7* double mutants (Figure 3C and D). Because the *act2/7* double mutant exhibited severe growth retardation, preventing direct comparison at the same time point, wild-type plants were observed at 5 d after germination (DAG), whereas the mutant was analyzed at 14 DAG. As previously reported,^10^ the *act2/7* double mutant produced almost completely hairless roots with abnormally swollen epidermal cells (Figure 3C), making it difficult to distinguish between hair-forming and non-hair-forming epidermal cell lineages; consequently, only a few root hair cells with a hair bulge could be identified. In such cells, nuclei were misplaced along the apical-basal axis (Figure 3D), as previously reported in *act7* mutants^7^; these observations collectively confirm the significant role of actin in polar nuclear positioning. Interestingly, the *act2/7* cells did not initiate the 2nd step but completed the 1st step, with the nucleus located adjacent to the inner membrane (Figure 3D). These results suggest that *ACT2* and *ACT7* are not essential for the 1st step of nuclear migration. To further determine the requirement of actin for the 1st step, it would be crucial to obtain the viable *act2/7/8* triple mutants by inducibly knocking out the *ACT8* gene in the *act2/7* double-knockout background.

**Figure 4.**
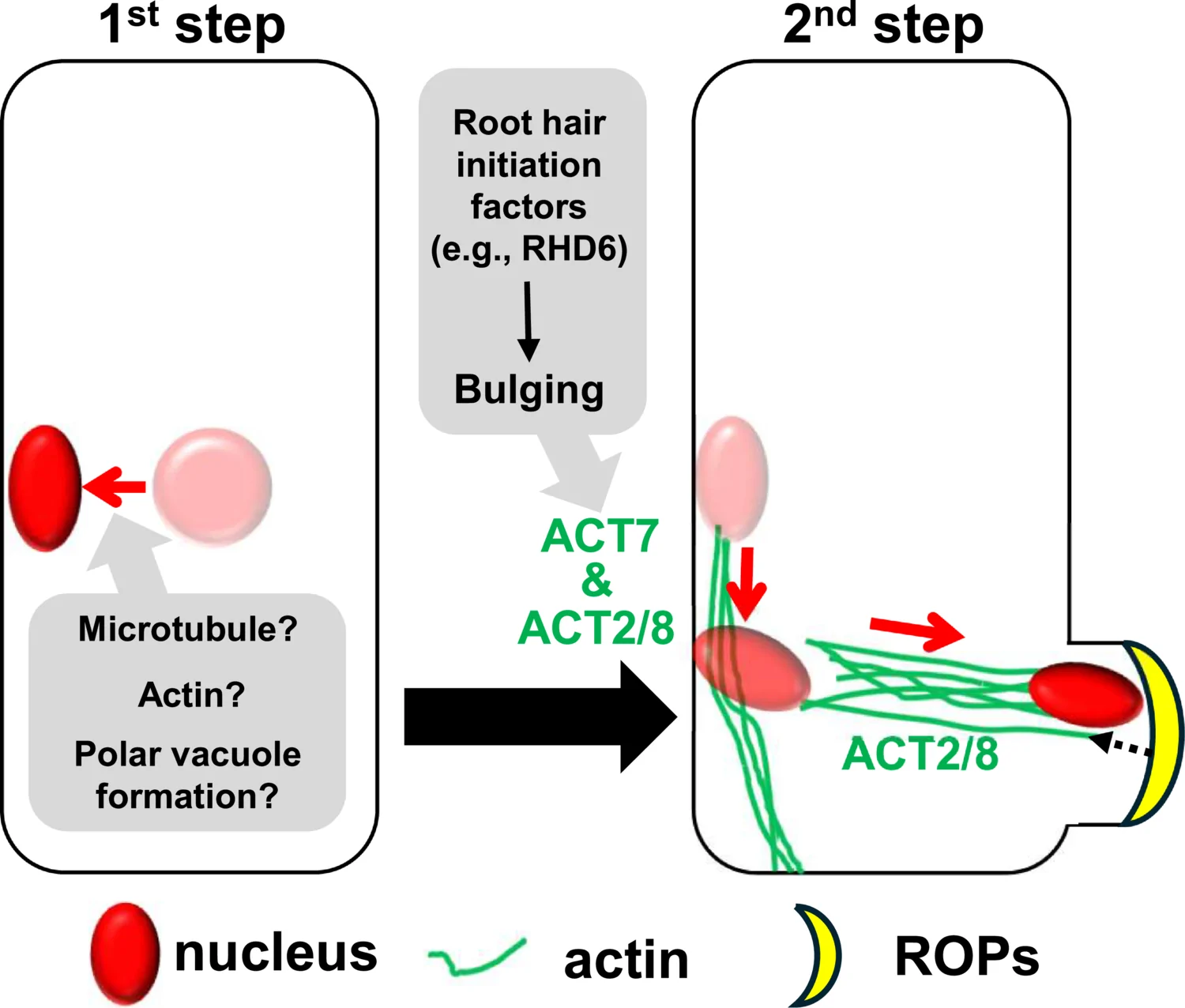
Schematic model illustrating the 1st and 2nd steps of nuclear migration during root hair initiation. While the 1st step of nuclear migration within root hair precursor cells initiates in a root hair initiation-independent manner, it remains unclear how the driving force behind this initial process is generated. For the subsequent 2nd step, bulge formation, involving RHD6 as a key regulator, is essential for its onset. Alternatively, RHD6 might trigger the onset of the 2nd step, independently of bulge formation.

There are two alternative explanations for the unexpected finding that *act* mutants did not exhibit obvious abnormalities during the 1st step. First, the driving force behind the 1st step is provided by MTs, but not by actin (Figure 4). Typically, nuclear movement is driven by actin in plant cells. However, this is not always the case. For instance, during the asymmetric cell division of the *Arabidopsis* stomatal lineage, the nucleus moves twice, the first of which has been reported to be dependent on MTs.^19^ More specifically, MTs are required to drive pre-mitotic nuclear migration from the one end of the cell to the other, which is essential for establishing the proper orientation and asymmetry of the cell division.^19^ By analogy, the 1st step in root hair cells might also be guided by MTs.

Second, the 1st step is not a cytoskeleton-dependent active event, but rather a passive process driven by the formation of a large central vacuole during root hair cell differentiation (Figure 4). In differentiated plant cells, a large central vacuole occupies the central area within the cell, pushing other organelles —including the nucleus— outward.^20^ As a consequence, the nucleus is typically located at the cell periphery in terminally differentiated plant cells.^21^ ^,^ ^22^ A previous study demonstrated that in mutants with a reduced vacuole size, the nuclear distribution was slightly shifted toward the outer lateral direction prior to the onset of the 2nd step,^7^ implying an association between vacuole formation and nuclear positioning in root hair cells. To ensure the inward movement, polar vacuole formation is essential; a large vacuole must preferentially form on the outer-membrane side within root hair cells and expand inward to push the nucleus toward the inner membrane. To test this hypothesis, it would be of interest to simultaneously live-image the process of vacuole formation and the 1st step of nuclear migration at a high spatiotemporal resolution.

A key cellular mechanism ensuring vigorous plant development is the dramatic enlargement of individual cells. In some cases, plant cells can expand their volume by more than a thousandfold, occasionally exceeding lengths of 1 mm^23^. To resolve the intracellular spatial constraints arising from such cell enlargement, relocating organelles to appropriate sites is a prerequisite, although its molecular basis remains unclear. To address this long-standing question, the multi-step, long-distance nuclear migration within root hair cells, as described in this study, provides a suitable model (Figure 4). Unraveling how each step of nuclear migration is precisely controlled, how these steps are temporally coordinated, and how the force driving each movement is generated will provide valuable insights into the molecular mechanisms optimizing organelle distribution within plant cells.

## Supplementary Material

Supplementary Material Supplementary_Figure_1.docx
