# Supplementary material for: Root hair initiation-dependent and -independent nuclear movements during root hair development in Arabidopsis thaliana
Source: Plant Signal Behav. 2026 Sep 17;21(1):2726829. doi: 10.1080/15592324.2026.2726829 (PMC13596882; doi:10.1080/15592324.2026.2726829)
Supplement: Supplementary file 1 [file KPSB_A_2726829_SM3976.docx]

**Figure S1. Wild-type hair cells that failed to form a hair bulge did not initiate the 2^nd^ step of nuclear migration.**

Representative images of a root hair precursor cell that failed to form a hair bulge in five-day-old roots of wild-type carrying H2B-tdTomato. Numbers shown in the panels indicate the time (min). Time 0 indicates the moment when the 1st step completed. The scale bar represents 10 μm.
